# Detecting arousals and sleep from respiratory inductance plethysmography

**DOI:** 10.1007/s11325-025-03325-z

**Published:** 2025-04-11

**Authors:** Eysteinn Finnsson, Ernir Erlingsson, Hlynur D. Hlynsson, Vaka Valsdóttir, Thora B. Sigmarsdottir, Eydís Arnardóttir, Scott A. Sands, Sigurður Æ Jónsson, Anna S. Islind, Jón S. Ágústsson

**Affiliations:** 1 Nox Research, Nox Medical, Katrínartún 2, 105 Reykjavík, Iceland; 2 Department of Computer Science, Reykjavik University, Reykjavik, Iceland; 3 Department of Computer Science, University of Iceland, Reykjavik, Iceland; 4 Division of Sleep and Circadian Disorders, Department of Medicine, Brigham and Women’s Hospital and Harvard Medical School, Boston, MA USA

**Keywords:** Machine learning, Home sleep testing, Sleep disorders, Sleep stage, Polysomnography

## Abstract

**Purpose:**

Accurately identifying sleep states (REM, NREM, and Wake) and brief awakenings (arousals) is essential for diagnosing sleep disorders. Polysomnography (PSG) is the gold standard for such assessments but is costly and requires overnight monitoring in a lab. Home sleep testing (HST) offers a more accessible alternative, relying primarily on breathing measurements but lacks electroencephalography, limiting its ability to evaluate sleep and arousals directly. This study evaluates a deep learning algorithm which determines sleep states and arousals from breathing signals.

**Methods:**

A novel deep learning algorithm was developed to classify sleep states and detect arousals from respiratory inductance plethysmography signals. Sleep states were predicted for 30-s intervals (one sleep epoch), while arousal probabilities were calculated at 1-s resolution. Validation was conducted on a clinical dataset of 1,299 adults with suspected sleep disorders. Performance was assessed at the epoch level for sensitivity and specificity, with agreement analyses for arousal index (ArI) and total sleep time (TST).

**Results:**

The algorithm achieved sensitivity and specificity of 77.9% and 96.2% for Wake, 93.9% and 80.4% for NREM, 80.5% and 98.2% for REM, and 66.1% and 86.7% for arousals. Bland–Altman analysis showed ArI limits of agreement ranging from - 32 to 24 events/hour (bias: - 4.4) and TST limits from - 47 to 64 min (bias: 8.0). Intraclass correlation was 0.74 for ArI and 0.91 for TST.

**Conclusion:**

The algorithm identifies sleep states and arousals from breathing signals with agreement comparable to established variability in manual scoring. These results highlight its potential to advance HST capabilities, enabling more accessible, cost-effective and reliable sleep diagnostics.

**Supplementary Information:**

The online version contains supplementary material available at 10.1007/s11325-025-03325-z.

## Introduction

During sleep, the brain transitions between different states: rapid eye movement (REM) sleep, non-REM (NREM) sleep (which encompasses N1, N2, and N3), and wakefulness. Monitoring these states is crucial for diagnosing and assessing various sleep disorders which may be characterized by disruption of the usual patterns and duration of NREM and REM sleep, or an unusual number of awakenings or arousals [1, 2]. This is typically done using polysomnography (PSG) equipped with electroencephalography (EEG), electrooculography (EOG), electromyography (EMG), electrocardiography (ECG), respiratory inductance plethysmography (RIP), nasal cannula, and pulse oximetry. However, PSG studies are expensive and often inconvenient for patients, leading to the adoption of home sleep testing (HST) as a more accessible alternative that focuses primarily on breathing and oxygenation signals. While HST is more affordable and easier to administer it has certain limitations. Notably, it cannot accurately assess sleep states and arousals due to the absence of EEG, EOG, and EMG measurements. These limitations reduce the effectiveness of HST in diagnosing sleep disorders other than sleep apnea. Moreover, it may result in underdiagnosis of sleep apnea in patients with reduced sleep time or those with a high proportion of hypopneas associated with arousals rather than desaturations [3, 4].

There are indications that sleep states and arousals can be inferred from breathing patterns. For instance, breathing during wakefulness is characterized by volitional overwrite of breathing control, including swallows, sniffs, breath-holds, speech, sighs, coughs, and body movements. In contrast, breathing is remarkably monotonous during NREM sleep, controlled primarily by metabolic demand [5, 6]. During REM sleep, breathing becomes more variable, both in terms of breath amplitude and timing. Additionally, REM sleep is characterized by a significant reduction in skeletal muscle tone, with breathing predominantly driven by the diaphragm [7, 8]. This loss of muscle tone affects the intercostal muscles, whose contraction expands the chest wall, leading to a distinctive breathing pattern during REM where the chest wall caves in during inspiration [6]. These variations in breathing dynamics during sleep reveal a strong coupling between the sleep states and physiological changes that are reflected in the breathing patterns. Indeed, previous work has indicated that there is information in the respiratory signal for discriminating between REM, NREM and Wake [9–12].

Likewise, there are reasons to think that arousals can also be detected by analyzing breathing patterns. Arousals are brief awakenings from sleep, often triggered by stimuli such as loud sounds or breathing obstructions. These arousals are associated with sympathetic activation within the autonomic nervous system, leading to increases in heart rate and blood pressure [13]. Additionally, ventilation dynamics are altered following arousal through both chemical and state-related mechanisms [6]. Accompanying arousals is an increased activation of skeletal muscles, including the engagement of the upper airway dilator muscles [6, 14]. This muscle activation plays a crucial role in resolving upper airway obstructions, particularly in the context of sleep apnea [15]. Arousals have further been shown to cause a transient increase in ventilation, a gasp-like reflex, that is called the ventilatory response to arousal (VRA). This reflex is independent of metabolic demand and occurs even in the absence of upper airway obstruction (e.g., apnea and hypopnea) [16–20]. Research on auditory-induced arousals demonstrated that in addition to an increase in ventilation, the VRA is accompanied by a decrease in inspiratory duration and remains consistent across different sleep states [16].

To measure breathing movements of the chest wall and abdomen, both PSG and HST rely on RIP technology. The American Academy of Sleep Medicine (AASM) recommends using RIP belts for detecting the presence of respiratory effort and as a secondary flow sensor to the nasal cannula [2]. High-quality RIP belts have been shown to provide a reliable measure of airflow, with strong correlation to nasal cannula readings [21]. The ability of RIP to simultaneously measure the dynamics of chest wall movements and airflow means that it captures all the relevant physiology required to assess sleep states and arousals from breathing.

In this paper, we apply artificial intelligence (AI) to infer the relationship between sleep and breathing, developing an algorithm that detects sleep states and arousals solely from breathing. This enables subsequent calculation of total sleep time (TST) as well as arousal index (ArI)—an indicator of sleep fragmentation—from HST, significantly expanding its scope and efficacy. This innovative approach has the potential to transform HST by providing a comprehensive assessment of sleep, while simultaneously paving the way towards more accurate and accessible diagnosis of sleep disorders.

## Materials and methods

Nox BodySleep 2.0 (NBS2) is an AI algorithm designed to determine sleep states and detect arousals using abdominal and thoracic RIP signals. The input signals are processed by a neural network, which assigns probabilities to each sleep state as well as to the likelihood of an arousal. An extensive dataset was used to assess the algorithm's efficacy, with careful attention to ensure that the subjects and data reflect the algorithm's intended application in standard clinical settings.

NBS2 is designed to aid in the scoring of home sleep tests that include no electrophysiological signals (e.g., EEG, EOG, and EMG). However, to rigorously evaluate NBS2, it was necessary to compare it with"gold standard"sleep and arousal scoring, which requires in-laboratory PSG recordings with high-quality electrophysiological signals. Accordingly, in the current study, PSG recordings were manually scored using all available signals while only RIP data were input to NBS2 to provide AI scoring for comparison, see Table 1. Of note, since our goal was to discriminate between three distinct states, namely Wake, REM and NREM, the gold standard labels for N1, N2, and N3 were combined into a single NREM state.

**Table 1:**
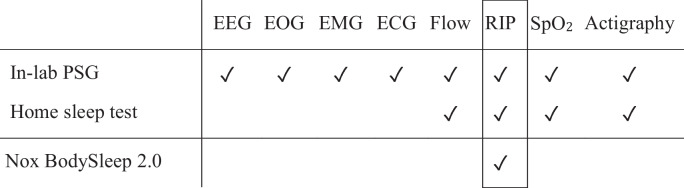
Comparison of signals recorded during in-laboratory polysomnography (PSG) and home sleep tests, along with the required inputs for the current algorithm under evaluation

^*^ Electroencephalography (EEG), electrooculography (EOG), electromyography (EMG), electrocardiography (ECG), airflow (Flow), respiratory inductance plethysmography (RIP), oxygen saturation (SpO_2_)

### Algorithm development

The NBS2 algorithm was developed using a dataset of PSG recordings from clinical and research settings across seven sites in Europe, Asia, and the United States. In total, 3,185 recordings were used: 2,216 for training, 482 for tuning, and 487 for testing. Note that the results presented in this study are based on an entirely separate dataset, unrelated to those used for algorithm training and development.

Training was performed using cross-entropy loss, optimized with the AdamW method as implemented in TensorFlow. Overfitting was monitored and the following regularization techniques were applied: spatial dropout and batch normalization. Data augmentation was employed which involved scaling the input signals and introducing random temporal shifts. Training samples were selected with a 50% probability of containing a scored arousal.

### Participants

A large set of in-lab PSG recordings were used to comparatively evaluate the algorithm’s performance. The PSG studies were drawn from dataset provided by FusionSleep clinics in Atlanta, Georgia, USA, between 2020 and 2023 (inclusive). This dataset is representative of the algorithm’s intended use in standard clinical practice in sleep clinics and hospitals, ensuring that the comparative results accurately reflect its real-world performance. Data was recorded using the Nox A1 sleep recorder (Nox Medical, Iceland). All recordings underwent standardized quality control and were scored by qualified sleep technologists according to the AASM manual for scoring sleep and associated events [2]. Finally, the scoring was reviewed and approved by a medical doctor.

The PSG recordings used for evaluation were selected based on predefined inclusion criteria, requiring that each study be manually scored, conducted during nighttime, and include an adult participant (≥ 18 years old). Additionally, both RIP belts had to be connected for at least half of the recorded sleep time. Studies not meeting these criteria were excluded from the analysis. No information was available on subject’s medication use or preexisting health conditions for this study.

### Algorithm description

**Fig. 1:**
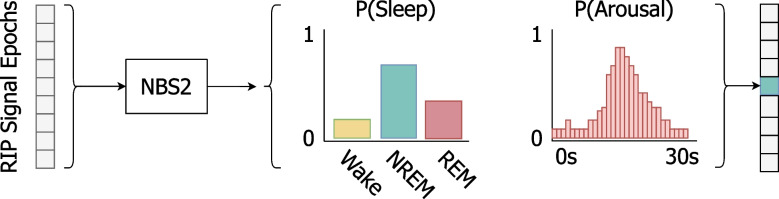
Overview of the Nox BodySleep 2.0 (NBS2) algorithm. The input consists of nine consecutive 30 s respiratory inductance plethysmography (RIP) signal epochs. NBS2 predicts the probability of sleep states (Wake, NREM, REM) and arousal (1 s resolution) for the center epoch. Sleep state is scored by selecting the state with the highest probability. An arousal is scored using a rule-based post-processing step, which requires that the arousal probability exceeds a specific threshold for at least 3 s. The output probabilities shown in the figure are illustrative and do not represent actual algorithm outputs

**Fig. 2:**
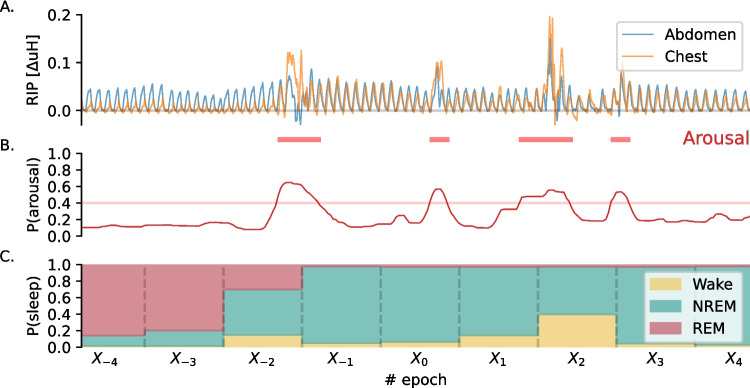
The algorithm analyses 4.5 min data segments at a time. Subplot A shows breathing movements recorded by two respiratory inductance plethysmography (RIP) belts placed around the abdomen and chest, which serve as inputs to the algorithm. Subplots B and C display the neural-network outputs: arousal probabilities and sleep state probabilities. Arousals are marked with red squares when the arousal probability exceeds a threshold for at least 3 s. The sleep state prediction in subplot C is presented as a hypnodensity graph [53], predicting the likelihood of each sleep state—Wake, NREM, or REM—across epochs. The depicted window of data captures a transition from REM sleep, characterized by reduced chest wall movement, to NREM sleep, where chest movement increases. This transition is associated with arousals and a higher probability of wakefulness

NBS2 requires only the abdomen and thorax RIP belt inductance signals as input. These signals are sampled at 25 Hz with a bandwidth of 0–12.5 Hz, measuring the loop inductance of the wire in each belt encircling the patient. A nonlinear adaptive filter is used to remove the baseline, gradually suppressing frequencies below 0.1 Hz while adapting to larger changes, such as patient movement. Nine consecutive signal epochs (where each epoch is a 30 s temporal window) are employed to process each target epoch, with four epochs preceding and four epochs following the target, as depicted in Fig. 1. For each of these epochs, there are two target outputs: 1) The sleep state in each epoch is derived according to the highest probability, followed by a holistic regression analysis that refines it, i.e., smooths consecutive sleep state outputs, which is a common regularization technique for time-series data [22]; 2) The algorithm computes the probability of an arousal event for each second within the target epoch. An arousal is subsequently identified if at least three consecutive seconds exceed a predefined threshold, in which case an arousal is scored along with its duration, see Fig. 2.

The neural network architecture is based on temporal convolutional networks (TCN), following the suggestions made by S. Bai et al. [23] to improve the performance of sequence modelling tasks. The architecture employs residual connections, regularization, and activation functions, to form a chain of residual blocks [24–26]. A schematic of model architecture is available in the supplementary materials (Figures S1-S2).

TCNs possess several features well suited to the task at hand. They efficiently handle long-range dependencies using dilated convolutions, effectively capturing the necessary context surrounding the window of interest. Furthermore, their ability to extract time-invariant features is particularly important for precisely localizing sleep-related events like arousals. Using a multi-task learning approach, we train a single model to handle both arousal detection and sleep state classification, enabling shared feature learning with separate outputs for each task (see Figure S2. in the supplementary materials).

### Statistical analysis

To evaluate the NBS2 algorithm's performance, we use both epoch-level agreement metrics and patient-level measures. Sensitivity, specificity, accuracy, and F1 score, are calculated to assess epoch-level agreement between NBS2 outputs and manual scoring. Additionally, Cohen’s Kappa is computed to quantify agreement, corrected for chance. Epochs containing manually scored signal artifacts are excluded from analysis. For patient-level measures, specifically TST and ArI, agreement are evaluated using Bland–Altman analysis and intraclass correlation coefficients (ICC).

Epoch-level sensitivity, specificity, accuracy, and F1 score are defined as follows: sensitivity = TP/(TP + FN), specificity = TN/(TN + FP), accuracy = (TP + TN)/(TP + TN + FP + FN), and F1 score = 2 TP/(2 TP + FP + FN) where TP, TN, FP, and FN denote true positives, true negatives, false positives, and false negatives, respectively. Sensitivity, specificity, accuracy, and F1 score, are calculated with 95% confidence intervals. The sleep state metrics are calculated for each class using a one-vs-rest method, while arousal epochs are identified based on their epoch presence or absence in either NBS2 or the manual scoring. Arousal detection performance is also analyzed by REM and NREM sleep states. Epoch-level measures of arousal detection are selected for comparison of medical devices through their FDA 510(k) summaries [27–29].

For patient-level measures, Bland–Altman analysis is used to calculate mean difference (bias) and limits of agreement (LoA), both with 95% confidence intervals. Similarly, ICC is computed, with corresponding 95% confidence intervals. Additionally, the algorithm's arousal scoring performance is evaluated across obstructive sleep apnea (OSA) severity levels, specifically examining ArI bias, LoA and ICC within defined apnea–hypopnea index (AHI) groups: normal (AHI < 5), mild (5 ≤ AHI < 15), moderate (15 ≤ AHI < 30), and severe (AHI ≥ 30).

In a supplementary analysis, all epoch- and patient-level measures are provided for each OSA severity group, as well as for periodic limb movement of sleep index (PLMSI) subgroups (PLMSI < 15 and PLMSI ≥ 15).

All confidence intervals are determined via bootstrapping with 10,000 iterations.

## Results

After applying the exclusion criteria to the initial pool of 1829 studies, the final validation dataset comprised 1,299 sleep studies, including 616 male subjects, 673 female subjects, and 10 subjects with unspecified gender (Table 2 provides additional anthropometric data). Overall, the RIP signal connectivity was good, with 98% of sleep studies exceeding 95% connectivity throughout the recording period, including initial sensor setup and end-of-study removal.

**Table 2:** Subject characteristics

Characteristic	Value	Unit
Age (Mean ± SD)	48.6 ± 17.0	years
Sex, N (M:F:unknown)	616:673:10	count
BMI (Mean ± SD)	29.5 ± 7.8	kg/m^2^
AHI (Mean ± SD)	26.9 ± 24.9	events/hour
AHI < 5	118	count
5 ≤ AHI < 15	416	count
15 ≤ AHI < 30	344	count
AHI ≥ 30	421	count
PLMSI (Mean ± SD)	2.2 ± 9.2	events/hour

^*^ Body Mass Index (BMI), Apnea Hypopnea Index (AHI), Periodic Limb Movement of Sleep Index (PLMSI)

Table 3 presents epoch-level sensitivity, specificity, accuracy, and F1 score, along with their respective 95% confidence intervals. Additionally, Table S1 in the supplementary materials shows the distribution of performance across the study population. Detailed confusion matrices are provided in Tables 4 and 5. In Table 4, the classification of manually scored epochs by the NBS2 algorithm is displayed. The results indicate that NBS2 accurately classifies 78% of Wake epochs, though it misclassifies 21% of these as NREM sleep. NBS2 correctly scores 94% of NREM epochs and 80% of the REM epochs. Of the REM epochs that were manually scored as REM, NBS2 misclassified 18% as NREM. Cohen’s Kappa value for overall sleep-state agreement was 0.77. Table 5 shows that NBS2 correctly identifies arousals in 66% of the epochs with a manually scored arousal and appropriately avoids scoring arousals in 87% of the epochs where no arousal was manually scored. Cohen’s Kappa value for overall arousal scoring agreement was 0.51.

**Table 3:** Epoch-level performance of the algorithm under investigation compared to manual scoring, presented in terms of sensitivity, specificity, accuracy, and F1 score. Arousal detection performance is further stratified by NREM and REM sleep states

	Sensitivity % [95% CI]	Specificity % [95% CI]	Accuracy % [95% CI]	F1 Score % [95% CI]
*Sleep State Classification*				
Wake	77.9 [76.8, 78.9]	96.2 [95.9, 96.5]	92.4 [92.1, 92.7]	81.0 [80.2, 81.8]
NREM	93.9 [93.5, 94.3]	80.4 [79.7, 81.2]	89.3 [89.0, 89.6]	92.1 [91.8, 92.3]
REM	80.5 [79.3, 81.6]	98.2 [98.1, 98.3]	95.9 [95.7, 96.1]	83.7 [83.0, 84.4]
*Arousal Classification*				
Arousal	66.1 [64.9, 67.2]	86.7 [86.2, 87.2]	81.7 [81.3, 82.1]	63.6 [62.7, 64.5]
*Arousal Classification by sleep state*				
Arousal | NREM	64.0 [62.7, 65.3]	90.9 [90.4, 91.4]	83.0 [82.6, 83.5]	68.8 [67.8, 69.8]
Arousal | REM	66.8 [65.3, 68.3]	86.9 [86.2, 87.6]	82.3 [81.7, 82.8]	63.5 [62.3, 64.5]

**Table 4:** The normalized epoch-level confusion matrix for algorithm-classified versus manually classified sleep states, with the total number of scored epochs in the far-right column

		Algorithm prediction			
		Wake	NREM	REM	# Epochs
Manual scoring	Wake	78%	21%	1%	216,260
	NREM	4%	94%	2%	684,101
	REM	2%	18%	80%	136,239

**Table 5:** Normalized epoch-level confusion matrix for algorithm-classified versus manually classified arousal scoring, with the total number of scored epochs in the far-right column

		Algorithm prediction		
		Positive	Negative	# Epochs
Manual scoring	Positive	66%	34%	250,773
	Negative	13%	87%	786,033

**Fig. 3:**
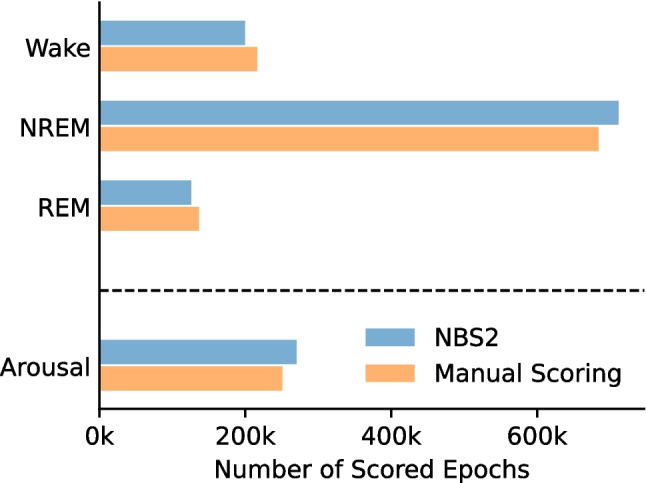
Comparison of the total number of epochs scored as Wake, NREM, REM, and those with arousals, between Nox BodySleep 2.0 (NBS2) and manual polysomnography scoring

Figure 3 compares the distribution of sleep states and arousal events between the NBS2 algorithm and manual PSG scoring, showing that NBS2 produces similar numbers of Wake, NREM, and REM sleep states, as well as a comparable number of arousal events, across the entire dataset. On average, arousals scored by NBS2 were 1.9 s longer than those scored manually. However, the total number of arousals identified by each approach was nearly identical, with NBS2 detecting 241,817 arousals and manual scoring identifying 241,377 see Figure S3 in the supplementary materials.

**Fig. 4:**
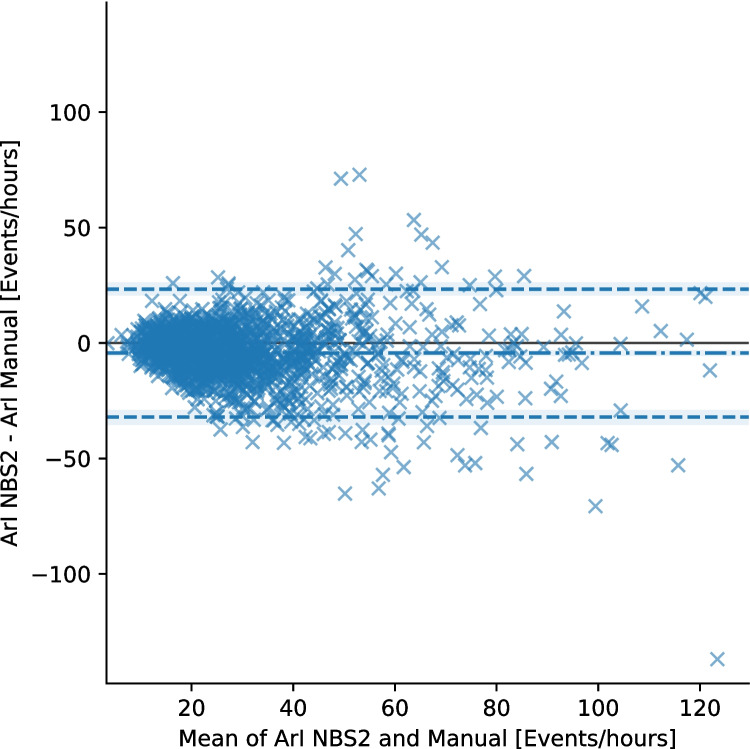
Bland–Altman plot showing the agreement between polysomnography scoring and Nox BodySleep 2.0 (NBS2) for Arousal Index (ArI). The plot includes the mean difference (bias, dot-dash line) and 95% limits of agreement (dashed blue lines). Each data point represents a single sleep study. The 95% confidence intervals for the bias and limits of agreement are shown as shaded regions

**Fig. 5:**
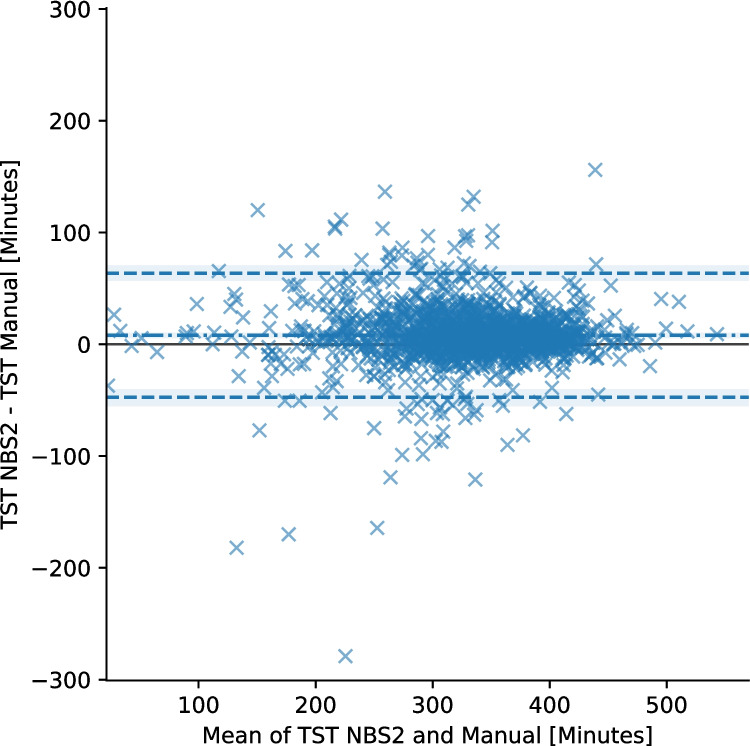
Bland–Altman plot showing the agreement between manual polysomnography scoring and Nox BodySleep 2.0 (NBS2) for total sleep time (TST). The plot includes the mean difference (bias, dot-dash line) and 95% limits of agreement (dashed blue lines). Each data point represents a single sleep study. The 95% confidence intervals for the bias and limits of agreement are shown as shaded regions

The agreement between NBS2 and manual scoring for both ArI and TST is assessed using Bland–Altman analysis of bias and LoA. For ArI, the analysis showed a bias of − 4.4 (95% CI: − 5.2, − 3.6) events/hour. The 95% LoA ranged from − 32 (95% CI: − 35, − 29) to 24 (95% CI: 21, 25) events/hour. Proportionality bias with slope − 0.127 (*p* < 0.05) was found. Log transformed Bland–Altman plot is available in Figure S4 in supplementary materials. For TST, the bias is 8.0 (95% CI: 6.5, 9.5) minutes, with 95% LoA spanning from − 47 (95% CI: − 54, − 41) to 64 (95% CI: 57, 69) minutes. See Bland–Altman plots in Figs. 4 and 5. ICC for ArI is 0.74 (95% CI: 0.69, 0.77) and for TST, it is 0.91 (95% CI: 0.89, 0.93).

In evaluating NBS2's performance in scoring arousals across OSA severity levels we observed consistent results. Here, LoA is shown as 1.96 times the standard deviation of the error. For patients without OSA (AHI < 5; *n* = 118), the arousal index showed a bias of − 3.63 events/hour, a LoA of 20.22 events/hour, and an ICC of 0.68 (95% CI: 0.57, 0.78). In mild OSA (5 ≤ AHI < 15; *n* = 416), the bias was − 3.08 events/hour with a LoA of 19.83 events/hour and an ICC of 0.67 (95% CI: 0.57, 0.76). Moderate OSA (15 ≤ AHI < 30; *n* = 344) had a bias of − 3.61 events/hour, a LoA of 21.34 events/hour, and an ICC of 0.54 (95% CI: 0.42, 0.66). In severe OSA (AHI ≥ 30; *n* = 421), the bias was higher at − 6.46 events/hour, with a LoA of 37.80 events/hour and an ICC of 0.62 (95% CI: 0.53, 0.69). A more detailed stratification of results across OSA severity levels as well as PLMSI subgroups (PLMSI ≥ 15, *n* = 51) can be found in Tables S2-S25 in the supplementary materials.

Additionally, each arousal scored by NBS2 was classified as either respiratory-related or not. An arousal was considered respiratory-related if a manually scored respiratory event (apnea or hypopnea) occurred within the 15 s preceding it. The proportion of respiratory-related arousals varied widely across the study population, ranging from 10 to 90% (median: 49%, central 95% range). This broad distribution underscores the algorithm's ability to detect arousals beyond those linked to respiratory events, effectively capturing patients with predominantly non-respiratory arousals as well as those with predominantly respiratory-related arousals.

## Discussion

This study introduces an innovative algorithm that utilizes RIP signals, analyzed through a novel AI architecture, to classify sleep states and arousals. We have demonstrated that both sleep states and arousals can be derived from RIP signals, achieving performance comparable to that of human EEG-based scoring. ICC is commonly used to quantify variability in expert human scoring; for TST, reported ICC values are 0.82 [30] and 0.87 [31], and for ArI, values range from 0.41 [32] to 0.68 [33]. The current algorithm achieved an ICC of 0.91 for TST and 0.74 for ArI, indicating strong alignment with expert EEG-based scoring. Detecting sleep states and arousal events without relying on EEG represents a substantial advancement, with potential to improve the accuracy and accessibility of sleep disorder diagnoses via HST.

Previous studies on deriving sleep states from non-EEG signals have used various combinations of physiological data, including respiratory and cardiac signals [34], respiratory, cardiac, and movement signals [35], and respiratory and movement signals [11, 12]. Much research has also focused on peripheral cardiac function using photoplethysmography (PPG), often combined with peripheral arterial tonometry (PAT) and actigraphy [36–41]. RIP signals alone have also been successfully used to determine sleep states in infants [9, 10]. Our group previously utilized RIP and actigraphy to estimate sleep states [11]. Notably, NBS2 improves upon the earlier method while relying solely on RIP signals, achieving higher sensitivity and specificity for all three sleep states as well as reducing TST overestimation from 14 to 8 min.

Additionally, achieving automatic arousal detection from breathing signals represents a significant milestone, given the longstanding challenge of detecting arousal events from any PSG signal. However, recent advancements in deep learning methods have shown promise in overcoming this challenge [42]. Attempts have been made at arousal detection from non-EEG signals using ECG [43, 44], PPG combined with respiratory flow [45] and PAT [46]. A downside of using cardiogenic signals for sleep state and arousal detection is the potential for confounding effects due to cardiac conditions and medications [47, 48], which are common among patients with sleep disorders [49–51]. By focusing on RIP signals, our approach avoids some of these confounding factors, potentially leading to more accurate and reliable detection of sleep state and arousal events. This is particularly important for patient populations prone to sleep fragmentation, such as the elderly [52] and those with sleep-disordered breathing characterized by arousals rather than desaturations [3]. Future studies should investigate the broader implications of RIP-based sleep and arousal detection in these groups.

Nevertheless, this study is not without limitations. The design and quality of RIP belts is expected to impact the generalizability of our results [21]. Similarly, excessively filtered low bandwidth signals are likely to impact performance of the algorithm, potentially compromising the accuracy of sleep staging and arousal detection. Thus, the choice of hardware and the preprocessing of signals are critical considerations for the successful application of this method. Another limitation of the study is that the validation cohort might not fully express the diversity of patients with sleep disorders and various comorbidities. Future studies will need to validate the algorithm’s performance across more diverse populations to ensure its effectiveness and reliability across a wide range of patient groups. In addition, the current study did not assess the impact of NBS2 on diagnostic measures such as the AHI, which incorporates sleep state scoring through the TST and relies on precise arousal detection for scoring hypopneas that do not meet desaturation criteria.

In conclusion, this study makes a two-fold contribution. First, it demonstrates that information about sleep state and arousals can be accurately extracted from breathing signals. Second, it introduces a novel AI-driven algorithm for sleep state scoring and arousal detection using respiratory signals, which could greatly enhance the accuracy and accessibility of sleep disorder diagnoses through HST. Combined, these contributions represent a promising advancement in our understanding and evaluation of sleep disorders.

## Supplementary Information

Below is the link to the electronic supplementary material.

Supplementary file1 (DOCX 543 KB)

## Data Availability

The data used in this study is proprietary and subject to contractual obligations that do not permit the authors to share it beyond the scope of this publication.
