# Supplementary material for: Detecting arousals and sleep from respiratory inductance plethysmography
Source: Sleep Breath. 2025 Apr 11;29(2):155. doi: 10.1007/s11325-025-03325-z (PMC11991959; doi:10.1007/s11325-025-03325-z)
Supplement: Supplementary file 1 — Supplementary file1 (DOCX 543 KB) [file 11325_2025_3325_MOESM1_ESM.docx]

Detecting arousals and sleep from respiratory inductance plethysmography

Supplementary materials

Eysteinn Finnsson^1, 2, †^, Ernir Erlingsson^1, 3, †^, Hlynur D Hlynsson^1^, Vaka Valsdóttir^1^, Thora B Sigmarsdottir^1^, Eydís Arnardóttir^1^, Scott A Sands^4^, Sigurður Æ Jónsson^1^, Anna S Islind^2^, Jón S Ágústsson^1^

^1^Nox Research, Nox Medical, Reykjavik, Iceland

^2^Department of Computer Science, Reykjavik University, Reykjavik, Iceland

^3^Department of Computer Science, University of Iceland, Reykjavik, Iceland

^4^Division of Sleep and Circadian Disorders, Department of Medicine, Brigham and Women’s Hospital and Harvard Medical School, Boston, MA, USA

^†^These authors contributed equally

**Correspondence:**
eysteinnf@noxmedical.com
Nox Medical, Katrínartún 2, 105 Reykjavík, Iceland

## Model architecture


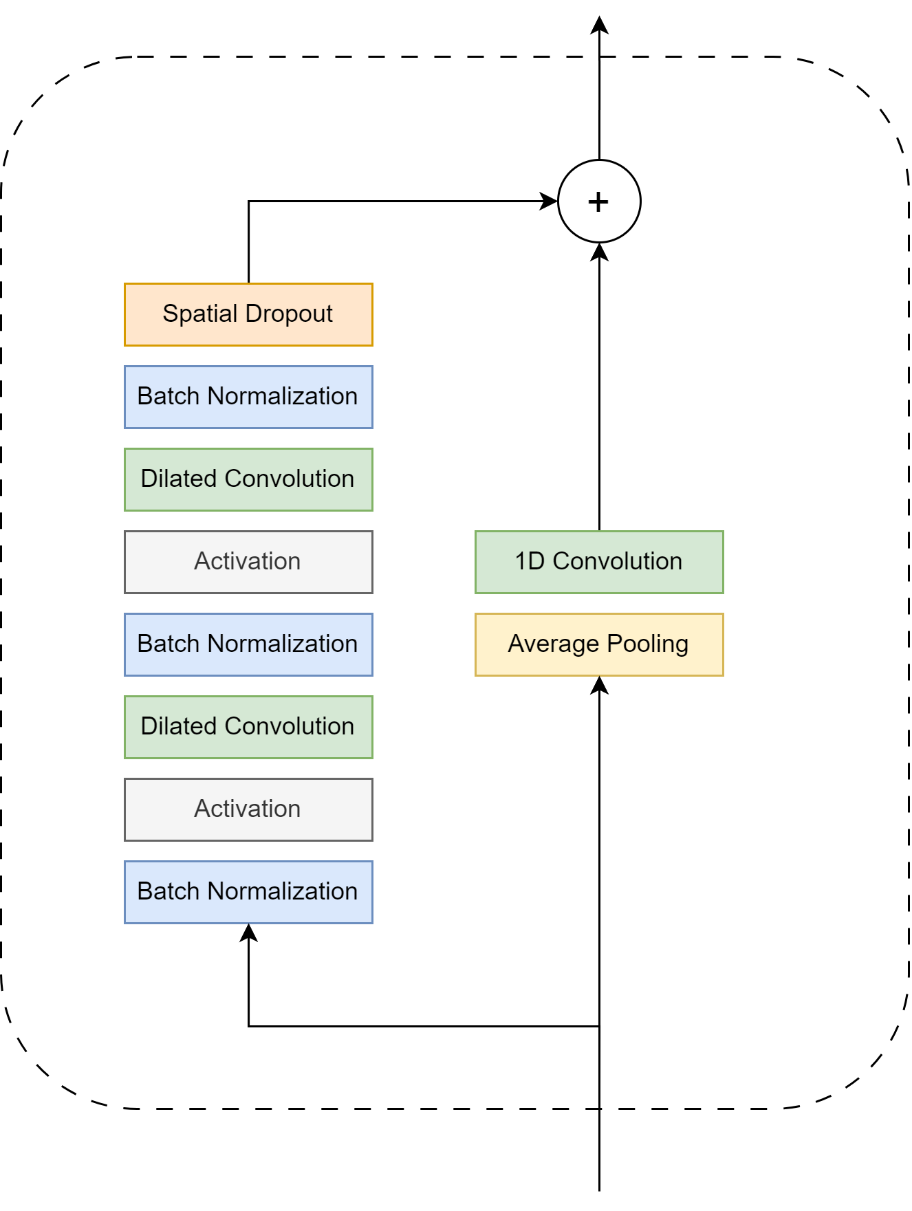


**Figure S1**: An example of a residual block used for Nox BodySleep 2.0 (NBS2) where raw time series data is processed through a series of steps. These steps include 1) Batch normalization, which normalizes across the respective data batch; 2) Activation function, NBS2 uses “Swish” which maps respective results to a range between a slightly negative value and one [1]; 3) Dilated Convolution, which uses spaced-out filters to capture long-range dependencies in time-series data; 4) Dropout, a common technique applied during training to increase the network´s robustness [2]. Additionally, the residual block includes a skip-connection, which is a pathway that effectively bypasses the main step sequence [3, 4]. The order of operations in our model was inspired by an architecture proposed by He et al. [5].


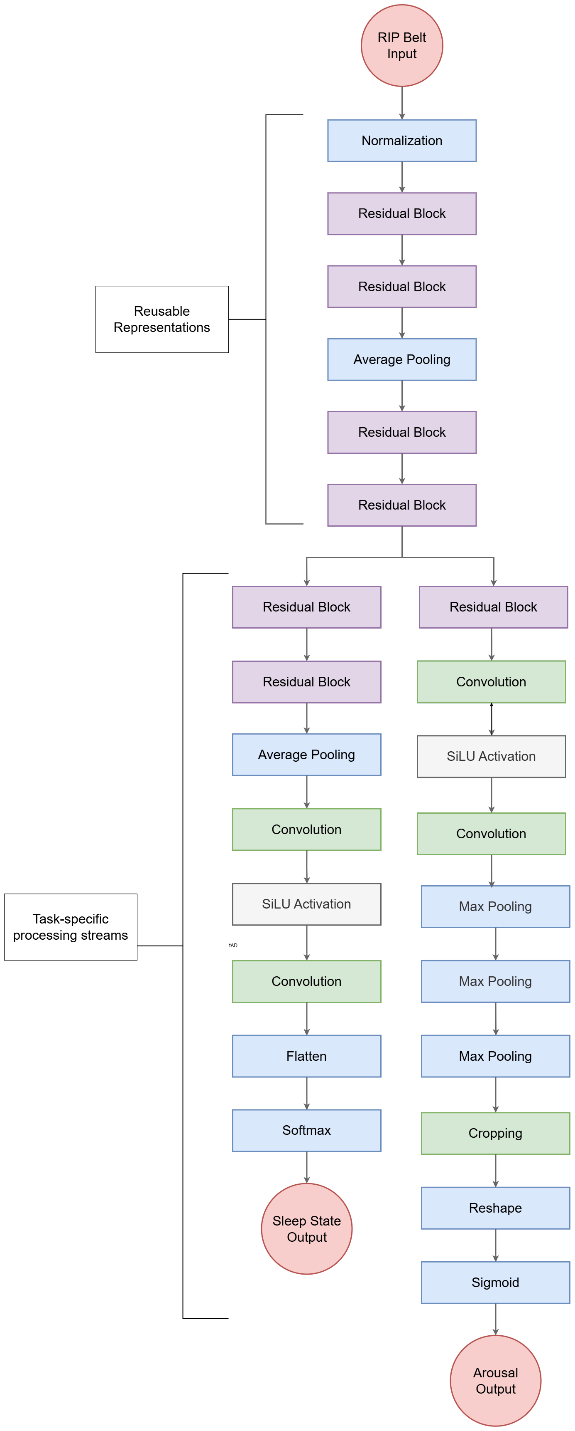


**Figure S2**: The model architecture for NBS2, where residual layers are illustrated in Figure S1. The RIP belt signals are provided as input (at 25Hz) to yield the sleep-state (Wake, REM, NREM) and arousal probabilities, where the former is returned for each sleep epoch and the latter on a second basis. The first six layers learn low-level features that are shared by both output branches. The arousal branch has two more layers than the sleep-state branch as it employs a different method to reduce the output to the desired temporal resolution; i.e., the arousal branch pools the data whereas the sleep-state branch flattens it and performs a Softmax activation.


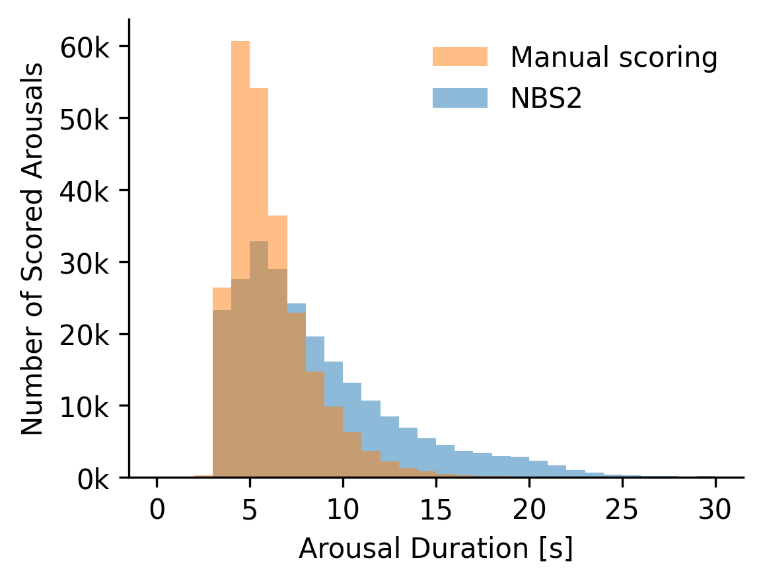


**Figure S3**: Distribution of arousal durations across the dataset (n=1299 patients). The histogram compares all manually scored arousals to all NBS2-scored arousals. In accordance with AASM scoring guidelines, neither approach scores arousals shorter than 3 seconds. NBS2 tends to score longer arousals (mean: 8.1 seconds, n=241,817) compared to manual scoring (mean: 6.2 seconds, n= 241,377). This discrepancy in arousal duration may be due to differences in scoring conventions between the training and validation datasets, which were sourced from different scoring sites.


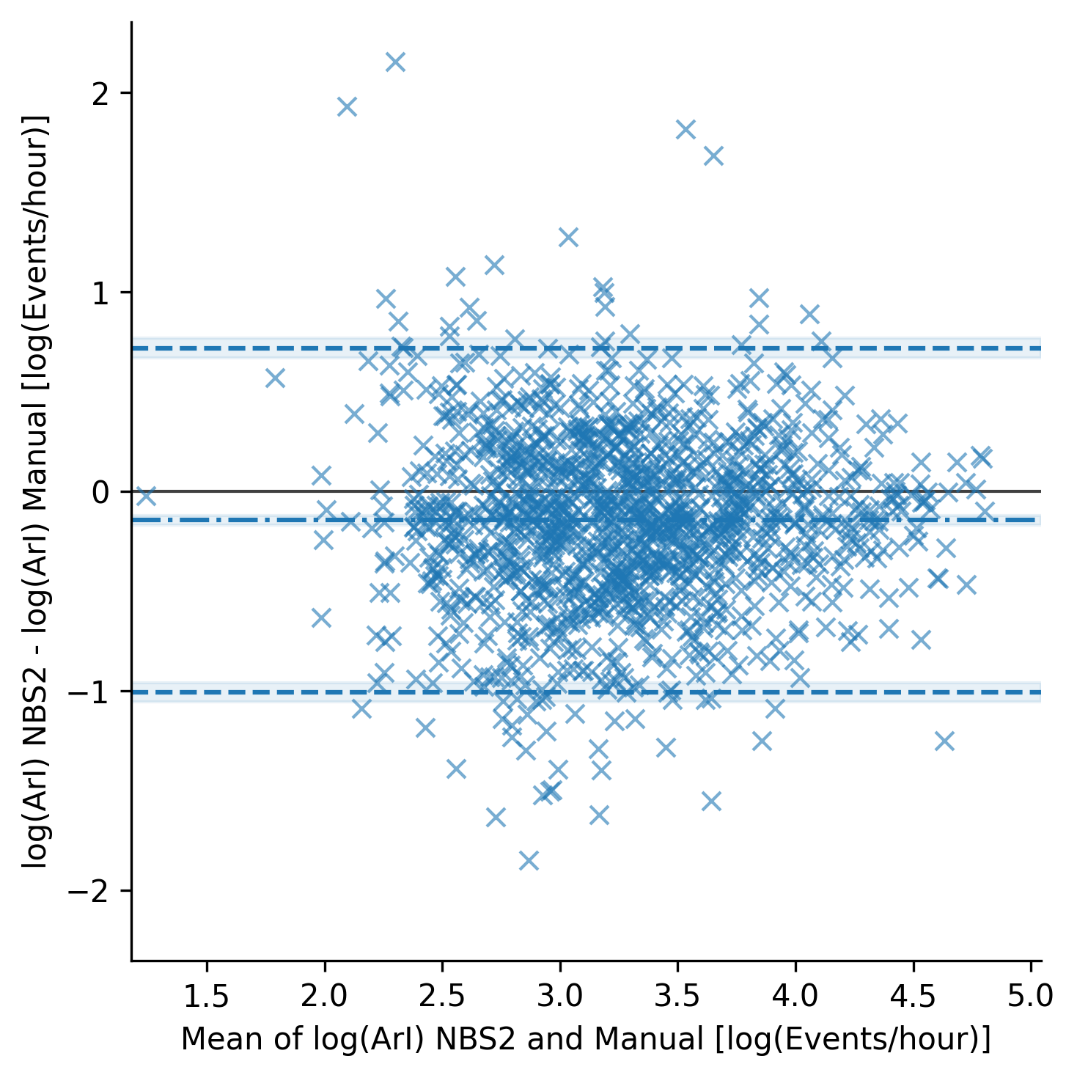


**Figure S4**: Bland-Altman plot of log-transformed Arousal Index (ArI) values, illustrating the agreement between Nox BodySleep 2.0 (NBS2) predicted and manually scored estimates.

## Stratified results

This section presents additional results for the Nox BodySleep 2.0 algorithm, showing performance metrics and confusion matrices segmented by apnea-hypopnea index (AHI) and periodic limb movement index (PLMSI) subgroups.

### Patient-level results statistics

Table S1: Distribution of algorithm performance across the study population (n=1299), reported as mean (standard deviation).

|  | Sensitivity % | Specificity % | Accuracy % | F1 Score % |
| --- | --- | --- | --- | --- |
| Wake | 75.0 (17.3) | 95.8 (7.2) | 92.3 (6.1) | 76.8 (14.7) |
| NREM | 93.6 (7.8) | 79.7 (13.8) | 89.3 (6.2) | 91.4 (6.8) |
| REM | 78.2 (22.9) | 98.2 (2.3) | 95.9 (3.2) | 76.6 (24.4) |
| Arousal | 64.3 (17.3) | 85.8 (10.1) | 81.6 (7.1) | 59.8 (13.3) |

### Epoch-level results segmented by AHI groups

Table S2: Epoch-level performance of the algorithm in detecting sleep states and arousals for the subset of patients with AHI < 5 events/hour (n=118). Results are reported as median [95% confidence interval] for sensitivity, specificity, accuracy, and F1 score.

|  | Sensitivity % | Specificity % | Accuracy % | F1 Score % |
| --- | --- | --- | --- | --- |
| Wake | 76.9 [72.9, 80.7] | 97.1 [96.3, 97.8] | 93.1 [92.0, 94.1] | 81.5 [78.5, 84.2] |
| NREM | 95.3 [94.4, 96.1] | 79.4 [76.7, 82.0] | 89.8 [88.7, 90.8] | 92.4 [91.6, 93.2] |
| REM | 79.8 [76.0, 83.1] | 98.6 [98.2, 98.9] | 95.8 [95.2, 96.4] | 84.9 [82.6, 86.9] |
| Arousal | 59.4 [56.2, 62.6] | 88.9 [87.6, 90.0] | 83.5 [82.3, 84.7] | 56.7 [54.0, 59.1] |

Table S3: Epoch-level performance of the algorithm in detecting sleep states and arousals for the subset of patients with 5 ≤ AHI < 15 events/hour (n=416). Results are reported as median [95% confidence interval] for sensitivity, specificity, accuracy, and F1 score.

|  | Sensitivity % | Specificity % | Accuracy % | F1 Score % |
| --- | --- | --- | --- | --- |
| Wake | 77.8 [75.7, 79.8] | 96.9 [96.5, 97.2] | 93.1 [92.6, 93.5] | 81.7 [80.3, 83.1] |
| NREM | 94.9 [94.4, 95.3] | 80.4 [78.8, 81.9] | 90.1 [89.5, 90.6] | 92.7 [92.3, 93.1] |
| REM | 80.4 [78.4, 82.2] | 98.3 [98.1, 98.5] | 96.0 [95.7, 96.2] | 84.1 [82.8, 85.2] |
| Arousal | 58.9 [57.3, 60.6] | 89.4 [88.8, 90.0] | 84.0 [83.5, 84.6] | 56.6 [55.4, 57.8] |

Table S4: Epoch-level performance of the algorithm in detecting sleep states and arousals for the subset of patients with 15 ≤ AHI < 30 events/hour (n=344). Results are reported as median [95% confidence interval] for sensitivity, specificity, accuracy, and F1 score.

|  | Sensitivity % | Specificity % | Accuracy % | F1 Score % |
| --- | --- | --- | --- | --- |
| Wake | 76.9 [74.9, 78.9] | 96.5 [95.7, 97.1] | 92.8 [92.1, 93.5] | 80.0 [78.3, 81.5] |
| NREM | 94.1 [93.1, 94.8] | 80.8 [79.4, 82.2] | 89.7 [89.0, 90.3] | 92.4 [91.9, 92.9] |
| REM | 82.5 [80.6, 84.3] | 98.1 [97.9, 98.4] | 95.9 [95.6, 96.2] | 85.3 [84.0, 86.4] |
| Arousal | 63.2 [61.3, 65.0] | 88.1 [87.3, 88.8] | 82.7 [82.0, 83.2] | 61.3 [60.1, 62.5] |

Table S5: Epoch-level performance of the algorithm in detecting sleep states and arousals for the subset of patients with AHI ≥ 30 events/hour (n=421). Results are reported as median [95% confidence interval] for sensitivity, specificity, accuracy, and F1 score.

|  | Sensitivity % | Specificity % | Accuracy % | F1 Score % |
| --- | --- | --- | --- | --- |
| Wake | 78.9 [77.3, 80.4] | 95.0 [94.4, 95.7] | 91.2 [90.5, 91.8] | 81.0 [79.6, 82.4] |
| NREM | 92.4 [91.6, 93.1] | 80.5 [79.2, 81.7] | 88.2 [87.5, 88.8] | 91.0 [90.4, 91.5] |
| REM | 78.7 [76.4, 80.8] | 98.0 [97.8, 98.3] | 95.8 [95.5, 96.1] | 81.2 [79.6, 82.6] |
| Arousal | 72.3 [70.4, 74.2] | 81.1 [79.9, 82.3] | 78.1 [77.3, 78.8] | 69.5 [68.1, 70.9] |

### Confusion matrices for sleep segmented by AHI groups

Table S6: Normalized epoch-level confusion matrix for algorithm-classified versus manually classified sleep states in the subset of patients with AHI < 5 events/hour (n=118). True labels are presented in rows, predicted labels in columns, and the total number of scored epochs is displayed in the far-right column.

|  | Wake | NREM | REM | # Epochs |
| --- | --- | --- | --- | --- |
| Wake | 77% | 22% | 1% | 19,366 |
| NREM | 3% | 95% | 2% | 63,989 |
| REM | 2% | 19% | 80% | 14,403 |

Table S7: Normalized epoch-level confusion matrix for algorithm-classified versus manually classified sleep states in the subset of patients with 5 ≤ AHI < 15 events/hour (n=416). True labels are presented in rows, predicted labels in columns, and the total number of scored epochs is displayed in the far-right column.

|  | Wake | NREM | REM | # Epochs |
| --- | --- | --- | --- | --- |
| Wake | 78% | 21% | 1% | 66,326 |
| NREM | 3% | 95% | 2% | 222,097 |
| REM | 2% | 18% | 80% | 44,077 |

Table S8: Normalized epoch-level confusion matrix for algorithm-classified versus manually classified sleep states in the subset of patients with 15 ≤ AHI < 30 events/hour (n=344). True labels are presented in rows, predicted labels in columns, and the total number of scored epochs is displayed in the far-right column.

|  | Wake | NREM | REM | # Epochs |
| --- | --- | --- | --- | --- |
| Wake | 77% | 22% | 1% | 51,426 |
| NREM | 4% | 94% | 2% | 184,720 |
| REM | 1% | 16% | 83% | 39,789 |

Table S9: Normalized epoch-level confusion matrix for algorithm-classified versus manually classified sleep states in the subset of patients with AHI ≥ 30 events/hour (n=421). True labels are presented in rows, predicted labels in columns, and the total number of scored epochs is displayed in the far-right column.

|  | Wake | NREM | REM | # Epochs |
| --- | --- | --- | --- | --- |
| Wake | 79% | 20% | 1% | 79,060 |
| NREM | 5% | 92% | 2% | 213,295 |
| REM | 2% | 19% | 79% | 37,958 |

### Confusion matrices for arousal segmented by AHI groups

Table S10: Normalized epoch-level confusion matrix for algorithm-classified versus manually classified arousals in the subset of patients with AHI < 5 events/hour (n=118). True labels are presented in rows, predicted labels in columns, and the total number of scored epochs is displayed in the far-right column.

|  | Arousal | No Arousal | # Epochs |
| --- | --- | --- | --- |
| Arousal | 59% | 41% | 17,754 |
| No Arousal | 11% | 89% | 80,030 |

Table S11: Normalized epoch-level confusion matrix for algorithm-classified versus manually classified arousals in the subset of patients with 5 ≤ AHI < 15 events/hour (n=416). True labels are presented in rows, predicted labels in columns, and the total number of scored epochs is displayed in the far-right column.

|  | Arousal | No Arousal | # Epochs |
| --- | --- | --- | --- |
| Arousal | 59% | 41% | 58,737 |
| No Arousal | 11% | 89% | 273,881 |

Table S12: Normalized epoch-level confusion matrix for algorithm-classified versus manually classified arousals in the subset of patients with 15 ≤ AHI < 30 events/hour (n=344). True labels are presented in rows, predicted labels in columns, and the total number of scored epochs is displayed in the far-right column.

|  | Arousal | No Arousal | # Epochs |
| --- | --- | --- | --- |
| Arousal | 63% | 37% | 60,070 |
| No Arousal | 12% | 88% | 215,930 |

Table S13: Normalized epoch-level confusion matrix for algorithm-classified versus manually classified arousals in the subset of patients with AHI ≥ 30 events/hour (n=421). True labels are presented in rows, predicted labels in columns, and the total number of scored epochs is displayed in the far-right column.

|  | Arousal | No Arousal | # Epochs |
| --- | --- | --- | --- |
| Arousal | 72% | 28% | 114,212 |
| No Arousal | 19% | 81% | 216,192 |

### Epoch-level results segmented by PLMSI groups

Table S14: Epoch-level performance of the algorithm in detecting sleep states and arousals for the subset of patients with PLMSI < 15 events/hour (n=1248). Results are reported as median [95% confidence interval] for sensitivity, specificity, accuracy, and F1 score.

|  | Sensitivity % | Specificity % | Accuracy % | F1 Score % |
| --- | --- | --- | --- | --- |
| Wake | 78.1 [77.0, 79.2] | 96.3 [95.9, 96.6] | 92.5 [92.2, 92.8] | 81.2 [80.3, 82.0] |
| NREM | 93.9 [93.5, 94.3] | 80.7 [79.9, 81.5] | 89.4 [89.1, 89.7] | 92.1 [91.9, 92.4] |
| REM | 80.9 [79.8, 81.9] | 98.2 [98.1, 98.3] | 95.9 [95.7, 96.1] | 83.9 [83.2, 84.6] |
| Arousal | 66.0 [64.8, 67.2] | 87.0 [86.5, 87.4] | 81.9 [81.5, 82.3] | 63.8 [62.9, 64.7] |

Table S15: Epoch-level performance of the algorithm in detecting sleep states and arousals for the subset of patients with PLMSI ≥ 15 events/hour (n=51). Results are reported as median [95% confidence interval] for sensitivity, specificity, accuracy, and F1 score.

|  | Sensitivity % | Specificity % | Accuracy % | F1 Score % |
| --- | --- | --- | --- | --- |
| Wake | 73.6 [67.0, 79.6] | 95.4 [93.4, 96.9] | 90.1 [87.8, 92.1] | 78.3 [73.6, 82.6] |
| NREM | 93.9 [91.7, 95.6] | 74.3 [69.3, 78.9] | 86.9 [84.7, 88.9] | 90.2 [88.3, 91.9] |
| REM | 68.6 [58.6, 77.1] | 98.6 [97.8, 99.2] | 95.2 [93.8, 96.4] | 76.1 [68.7, 81.8] |
| Arousal | 67.1 [61.6, 72.2] | 80.6 [77.3, 83.5] | 77.2 [75.2, 79.2] | 59.1 [55.9, 62.3] |

### Confusion matrices for sleep segmented by PLMSI groups

Table S16: Normalized epoch-level confusion matrix for algorithm-classified versus manually classified sleep states in the subset of patients with PLMSI < 15 events/hour (n=1248). True labels are presented in rows, predicted labels in columns, and the total number of scored epochs is displayed in the far-right column.

|  | Wake | NREM | REM | # Epochs |
| --- | --- | --- | --- | --- |
| Wake | 78% | 21% | 1% | 206,490 |
| NREM | 4% | 94% | 2% | 658,446 |
| REM | 2% | 17% | 81% | 131,814 |

Table S17: Normalized epoch-level confusion matrix for algorithm-classified versus manually classified sleep states in the subset of patients with PLMSI ≥ 15 events/hour (n=51). True labels are presented in rows, predicted labels in columns, and the total number of scored epochs is displayed in the far-right column.

|  | Wake | NREM | REM | # Epochs |
| --- | --- | --- | --- | --- |
| Wake | 74% | 24% | 2% | 9,688 |
| NREM | 5% | 94% | 1% | 25,655 |
| REM | 3% | 29% | 68% | 4,413 |

### Confusion matrices for arousal segmented by PLMSI groups

Table S18: Normalized epoch-level confusion matrix for algorithm-classified versus manually classified arousals in the subset of patients with PLMSI < 15 events/hour (n=1248). True labels are presented in rows, predicted labels in columns, and the total number of scored epochs is displayed in the far-right column.

|  | Arousal | No Arousal | # Epochs |
| --- | --- | --- | --- |
| Arousal | 66% | 34% | 240,989 |
| No Arousal | 13% | 87% | 756,026 |

Table S19: Normalized epoch-level confusion matrix for algorithm-classified versus manually classified arousals in the subset of patients with PLMSI ≥ 15 events/hour (n=51). True labels are presented in rows, predicted labels in columns, and the total number of scored epochs is displayed in the far-right column.

|  | Arousal | No Arousal | # Epochs |
| --- | --- | --- | --- |
| Arousal | 67% | 33% | 9,784 |
| No Arousal | 19% | 81% | 30,007 |

### Parameter results segmented by AHI groups

Table S20: Parameter results for patients with AHI < 5 events/hour (n=118). Values are reported as median [95% confidence interval]. Arousal Index is measured in events/hour, and Total Sleep Time in minutes. Agreement is assessed using the intraclass correlation coefficient (ICC, unitless) and Bland-Altman (BA) analysis.

|  | Arousal Index | Total Sleep Time |
| --- | --- | --- |
| ICC | 0.68 [0.57, 0.78] | 0.92 [0.87, 0.95] |
| BA bias | -3.62 [-5.55, -1.80] | 10.66 [5.69, 15.96] |
| BA aggrement | 20.26 [-23.88, 16.62] | 55.58 [-44.82, 66.29] |
| BA aggrement lower | -23.88 [-28.95, -18.92] | -44.82 [-56.68, -33.62] |
| BA aggrement upper | 16.62 [13.32, 19.89] | 66.29 [52.24, 80.71] |

Table S21: Parameter results for patients with 5 ≤ AHI < 15 events/hour (n=416). Values are reported as median [95% confidence interval]. Arousal Index is measured in events/hour, and Total Sleep Time in minutes. Agreement is assessed using the intraclass correlation coefficient (ICC, unitless) and Bland-Altman (BA) analysis.

|  | Arousal Index | Total Sleep Time |
| --- | --- | --- |
| ICC | 0.67 [0.57, 0.76] | 0.91 [0.88, 0.93] |
| BA bias | -3.07 [-4.07, -2.13] | 9.34 [6.85, 11.87] |
| BA aggrement | 19.87 [-22.92, 16.82] | 50.74 [-41.44, 60.10] |
| BA aggrement lower | -22.92 [-26.35, -20.31] | -41.44 [-47.96, -35.20] |
| BA aggrement upper | 16.82 [14.69, 19.30] | 60.10 [52.70, 68.24] |

Table S22: Parameter results for patients with 15 ≤ AHI < 30 events/hour (n=344). Values are reported as median [95% confidence interval]. Arousal Index is measured in events/hour, and Total Sleep Time in minutes. Agreement is assessed using the intraclass correlation coefficient (ICC, unitless) and Bland-Altman (BA) analysis.

|  | Arousal Index | Total Sleep Time |
| --- | --- | --- |
| ICC | 0.54 [0.41, 0.66] | 0.88 [0.81, 0.93] |
| BA bias | -3.60 [-4.76, -2.46] | 7.22 [3.85, 10.39] |
| BA aggrement | 21.30 [-24.93, 17.68] | 58.68 [-51.46, 66.04] |
| BA aggrement lower | -24.93 [-27.64, -22.31] | -51.46 [-72.11, -34.55] |
| BA aggrement upper | 17.68 [15.24, 20.57] | 66.04 [51.35, 82.64] |

Table S23: Parameter results for patients with AHI ≥ 30 events/hour (n=421). Values are reported as median [95% confidence interval]. Arousal Index is measured in events/hour, and Total Sleep Time in minutes. Agreement is assessed using the intraclass correlation coefficient (ICC, unitless) and Bland-Altman (BA) analysis.

|  | Arousal Index | Total Sleep Time |
| --- | --- | --- |
| ICC | 0.62 [0.53, 0.69] | 0.92 [0.89, 0.94] |
| BA bias | -6.44 [-8.33, -4.63] | 6.57 [3.79, 9.30] |
| BA aggrement | 37.84 [-44.26, 31.41] | 55.34 [-48.76, 61.94] |
| BA aggrement lower | -44.26 [-50.46, -39.30] | -48.76 [-58.72, -39.77] |
| BA aggrement upper | 31.41 [26.88, 36.67] | 61.94 [54.47, 70.00] |

### Parameter results segmented by PLMSI groups

Table S24: Parameter results for patients with PLMSI < 15 events/hour (n=1248). Values are reported as median [95% confidence interval]. Arousal Index is measured in events/hour, and Total Sleep Time in minutes. Agreement is assessed using the intraclass correlation coefficient (ICC, unitless) and Bland-Altman (BA) analysis.

|  | Arousal Index | Total Sleep Time |
| --- | --- | --- |
| ICC | 0.74 [0.69, 0.77] | 0.91 [0.89, 0.93] |
| BA bias | -4.62 [-5.40, -3.86] | 7.86 [6.30, 9.41] |
| BA aggrement | 27.19 [-31.83, 22.59] | 54.42 [-46.54, 62.37] |
| BA aggrement lower | -31.83 [-34.91, -29.30] | -46.54 [-54.10, -40.42] |
| BA aggrement upper | 22.59 [20.37, 25.24] | 62.37 [56.68, 68.78] |

Table S25: Parameter results for patients with PLMSI ≥ 15 events/hour (n=51). Values are reported as median [95% confidence interval]. Arousal Index is measured in events/hour, and Total Sleep Time in minutes. Agreement is assessed using the intraclass correlation coefficient (ICC, unitless) and Bland-Altman (BA) analysis.

|  | Arousal Index | Total Sleep Time |
| --- | --- | --- |
| ICC | 0.70 [0.42, 0.85] | 0.85 [0.73, 0.94] |
| BA bias | 1.93 [-2.63, 6.40] | 11.48 [1.64, 21.67] |
| BA aggrement | 31.88 [-29.91, 33.72] | 70.34 [-58.22, 81.79] |
| BA aggrement lower | -29.91 [-39.06, -21.26] | -58.22 [-85.18, -35.00] |
| BA aggrement upper | 33.72 [24.74, 43.79] | 81.79 [53.84, 108.84] |

## REFERENCES

1. Ramachandran P, Zoph B, Le QV (2017) Searching for Activation Functions

2. Srivastava N, Hinton G, Krizhevsky A, et al (2014) Dropout: A Simple Way to Prevent Neural Networks from Overfitting. Journal of Machine Learning Research 15:1929–1958

3. He K, Zhang X, Ren S, Sun J (2015) Deep Residual Learning for Image Recognition

4. He T, Zhang Z, Zhang H, et al (2019) Bag of Tricks for Image Classification with Convolutional Neural Networks

5. He K, Zhang X, Ren S, Sun J (2016) Identity Mappings in Deep Residual Networks
